# Identification and validation of redox-immune based prognostic signature for hepatocellular carcinoma

**DOI:** 10.7150/ijms.56289

**Published:** 2021-03-10

**Authors:** Kangsheng Tu, Jin Li, Huanye Mo, Yao Xian, Qiuran Xu, Xuelian Xiao

**Affiliations:** 1 Department of Hepatobiliary Surgery, The First Affiliated Hospital of Xi'an Jiaotong University, Xi'an 710061, China.; 2 Department of Shoulder and Elbow Surgery, Honghui Hospital, Xi'an Jiaotong University, Xi'an 710054, China.; 3 Department of Nutrition, The First Affiliated Hospital of Xi'an Jiaotong University, Xi'an 710061, China.; 4 Key Laboratory of Tumor Molecular Diagnosis and Individualized Medicine of Zhejiang Province, Zhejiang Provincial People's Hospital (People's Hospital of Hangzhou Medical College), Hangzhou 310014, China.

**Keywords:** hepatocellular carcinoma, redox, immune, prognosis

## Abstract

The intimate interaction between redox signaling and immunity has been widely revealed. However, the clinical application of relevant therapeutic is unavailable due to the absence of validated markers that stratify patients. Here, we identified novel biomarkers for prognosis prediction in hepatocellular carcinoma (HCC). Prognostic redox-immune-related genes for predicting overall survival (OS) of HCC were identified using datasets from TCGA, LIRI-JP, and GSE14520. LASSO Cox regression was employed to construct the signature model and generate a risk score in the TCGA cohort. The signature contained CDO1, G6PD, LDHA, GPD1L, PPARG, FABP4, CCL20, SPP1, RORC, HDAC1, STC2, HDGF, EPO, and IL18RAP. Patients in the high-risk group had a poor prognosis compared to the low-risk group. Univariate and multivariate Cox regressions identified this signature as an independent factor for predicting OS. Nomogram constructed by multiple clinical parameters showed good performance for predicting OS indicated by the c-index, the calibration curve, and AUC. GSEA showed that oxidoreductase activity and peroxisome-related metabolic pathways were enriched in the low-risk group, while glycolysis activity and hypoxia were higher in the high-risk group. Furthermore, immune profiles analysis showed that the immune score and stromal score were significantly decreased in the high-risk group in the TCGA cohort. There was a considerably lower infiltration of anti-tumor immune cells while a higher proportion of pro-tumor immune cells in silico. Immune markers were distinctly expressed between the subgroups, and redox-sensitive immunoregulatory biomarkers were at higher levels in the high-risk group. Altogether, we identified a redox-immune prognostic signature. A more severe redox perturbation-driven immunosuppressive environment in the high-risk group stratified by the signature may account for poor survival. This may provide a clue to the combined therapy targeting redox and immune in HCC.

## Introduction

Hepatocellular carcinoma (HCC), accounting for about 85% - 90% of primary liver cancer, is the third leading cause of cancer-related death 1, 2. Nearly half of the new cases and deaths occurred in China 1, 2. Surgical resection, ablative therapies, and transarterial chemoembolization are routine treatments for HCC 3. Advanced disease at diagnosis and high recurrence rate in post-operation emerge are the leading causes of poor prognosis of HCC 3. Recently, an immune therapy agent targeting programmed death 1 (PD1), nivolumab, has been approved to be applied in HCC therapy 4, while many patients showed poor response to this drug 5. A combination of immune therapy and other novel agents targeting redox signaling, angiogenesis, for instance, may improve the treatment efficacy 6, 7.

Oxidative stress is one of the cancer characteristics arising from an imbalance between the production of oxidative molecules and eliminating antioxidant components, which plays a crucial role in hepatocarcinogenesis 8, 9. Redox reactions at the thiol side chain of protein cysteine residues are considered a cellular signal transduction mode. It intertwines with the eight hallmarks of cancer to manipulate cell fate, such as cell proliferation angiogenesis, invasion, and metastasis 10. As the typical reactive species, a surplus of reactive oxygen species (ROS) is accumulated in cancer due to cancer cells' vigorous metabolism 11. Uncontrolled ROS causes DNA damage, genome instability, and gene mutation, resulting in the abnormal expression of oncogenes or tumor suppressor genes 10. Metabolic alterations are intimately coupled with redox perturbation in cancer, mainly involving conversion between oxidative phosphorylation (OXPHOS) and glycolysis, fatty acid oxidation, and energy metabolism 6, 12. In addition to the influence on cancer cells, redox signaling also plays a crucial role in controlling immunity 13. Redox homeostasis is essential to maintaining physiological immune responses, while imbalances in cellular redox status lead to an uncontrolled immune process covering innate and adaptative immune response 12, 14. As the first line of immune defense, neutrophils are recruited into the site with infection and release ROS to kill pathogens 15. Subsequently, monocytes migrate to the inflammatory site by chemotactic gradients and differentiate into either M1 or M2 macrophages 12. Polarization into M1 is accompanied by the production of massive ROS 12. ROS affects macrophages' metabolism since M1 depends more on glycolysis, while M2 leans to capitalize on OXPHOS to satisfy energy demand 16. In the adaptive immune response perspective, redox signaling is an essential player in regulating T cell activation, proliferation, and differentiation 13. Although there is an irrefutable relationship between redox state and immune response, few studies have concerned the comprehensive effect of them on cancer progression.

This study aimed to construct a predictive model for HCC prognosis based on redox and immune markers. The risk score was calculated according to the signature for risk stratification. We validated the model's elegant performance in predicting overall survival (OS) and shed light on the signature's potential value in stratifying patients and guiding combined treatments for HCC.

## Materials and methods

### Study cohorts and data collection

Data sets used in the study comprised three cohorts, which were TCGA-LIHC (n=368) from the cancer genome atlas (TCGA) database, LIRI-JP (n = 231) from the International Cancer Genome Consortium (ICGC) database, and GSE14520 (n = 242) from the Gene Expression Omnibus (GEO) database. Patients who met the criteria were enrolled: (1) histologically diagnosed as hepatocellular carcinoma; (2) available mRNA expression data; (3) available OS information. Average RNA expression was calculated instead of duplications, and genes with low abundance were discarded.

### Acquisition of redox-immune related genes profile

Redox-related genes (RRGs) were downloaded from the Molecular Signatures Database (https://www.gsea-msigdb.org/gsea/msigdb/index.jsp), and immune-related genes (IRGs) were obtained from the ImmPort Database (https://www.immport.org/home). Among 461 RRGs and 1811 IRGs, genes available in all of the three datasets were enrolled in the analysis subsequently.

### Construction of prognostic redox-immune related genes signature

Prognostic RRGs and IRGs were generated by univariate Cox regression analysis. The common genes were obtained by the intersection of prognostic genes in the three datasets using the Venn diagram (http://bioinformatics.psb.ugent.be/webtools/Venn/). The least absolute shrinkage and selection operator (LASSO) Cox regression model was further employed to identify the crucial signature genes from the 52 common genes and the corresponding coefficients of genes 17. LASSO Cox regression was performed using the “glmnet” package in R software, and ten-fold cross-validation and 1000 iterations were conducted to get the reliable result as much as possible 18. A prognostic model consisted of 14 genes was developed based on the individual risk score. Each patient's risk score was calculated according to the signature genes, i.e., risk score = ∑ (coefficient_i_ × expression of signature gene_i_). Principal component analysis (PCA) was executed to determine the clustering efficacy of the signature.

### Survival analysis

OS between patients with low risk and high risk was compared via Kaplan-Meier analysis using the “survival” package in R software. A receiver operator characteristic (ROC) curve was generated to evaluate the accuracy of the risk signature for OS prediction. Univariate Cox analyses were performed to identify potential prognostic factors, and multivariate Cox analyses were used to determine risk score as an independent risk factor for OS.

### Construction of nomogram

Multivariate Cox regression model was applied to construct the nomogram to predict 1-, 3-, 5-year OS. The calibration curve, c-index, and the area under the receiver operating characteristic curve (AUC) were used to assess the performance of the prognostic nomogram. The calibration curve was plotted using a bootstrap method with 1,000 resamples to compare the predicted OS with the observed OS. C-index was calculated to estimate the discrimination ability of the model. AUC was similar to c-index and a higher value of which indicated better prognostic value.

### Gene Set Enrichment Analysis (GSEA)

GSEA was performed to explore the GO and KEGG terms enriched in the different-risk groups in GSEA 4.1.0. The annotated gene sets were c5.all.v7.2.symbols.gmt and c2.cp.kegg.v7.2.symbols.gmt for GO and KEGG enrichment analyses, respectively. Gene set permutations were performed 1,000 times for each analysis.

### Tumor microenvironment analysis

Immune score and stromal score were estimated to infer the proportion of immune and stromal cells using the ESTIMATE method using the “estimate” package in R software 19. The abundance of 22 immune cells was estimated based on the LM22 signature by CIBERSORT (https://cibersort.stanford.edu/) 20. Twenty-two immune cell types included naive B cells, memory B cells, plasma cells, CD8+ T cells, naïve CD4+ T cells, resting memory CD4+ T cells, activated memory CD4+ T cells, follicular helper T cells, Tregs, γδ T cells, resting NK cells, activated NK cells, monocytes, M0 macrophages, M1 macrophages, M2 macrophages, resting DCs, activated DCs, resting mast cells, activated mast cells, eosinophils, and neutrophils.

### Statistical analysis

Statistical analyses were performed using R software (Version 4.0.2). Correlation analysis was accomplished by calculating the Spearman correlation test. Differences of relevant markers between subgroups were compared by non-parameters test. P < 0.05 was considered significantly different.

## Results

### Construction of redox-immune based signature

**Figure 1:**
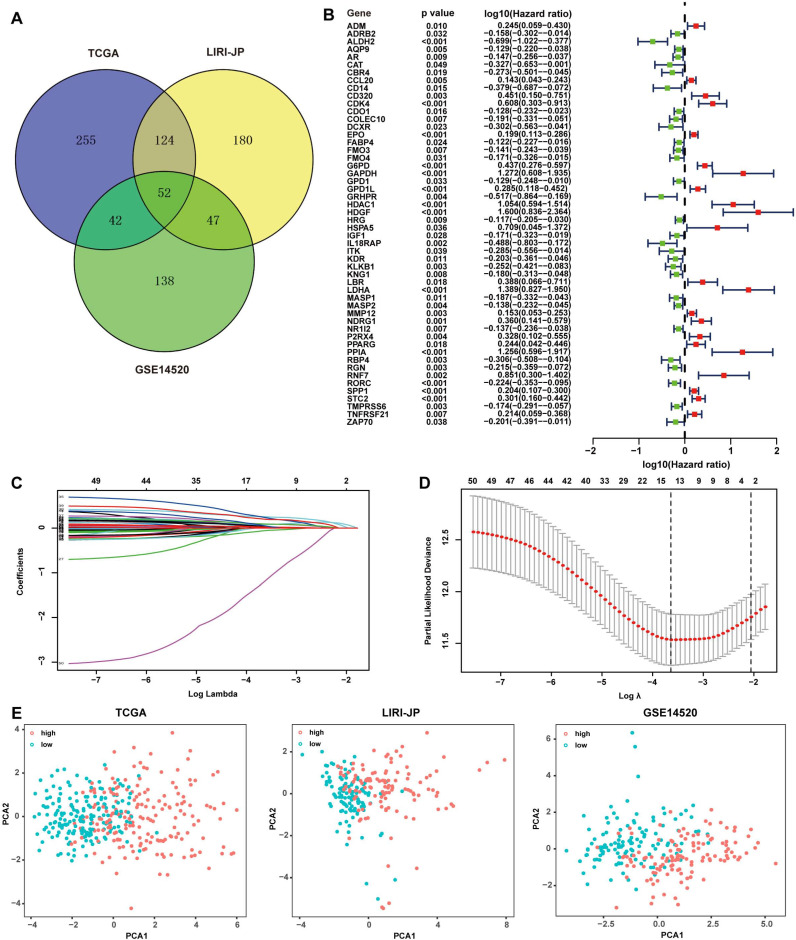
** Development of redox-immune-based gene signature for prognosis.** (A) Venn diagram revealing 52 common genes for prognosis in TCGA, LIRI-JP, and GSE14520 dataset. (B) Forest plot of univariable Cox proportional hazards for 52 redox-immune signature genes in TCGA. (C and D) LASSO Cox regression model was constructed from the 52 signature genes. The tuning parameter λ was calculated basing on the partial likelihood deviance with 10-fold cross-validation, and the coefficient was plotted against Log(λ). The 14 signature genes were identified according to the best fit profile. (E) PCA was based on the 14 signature genes classified by different risk in TCGA, LIRI-JP, and GSE14520 cohort, respectively.

The subjects contained 368 patients from TCGA, 231 patients from LIRI-JP, and 242 patients from GSE14520. 461 RRGs and 1811 IRGs were used for the construction of the prognostic model. Univariate Cox regression analyses were performed to excavate potential prognostic genes based on transcription profile, and 52 common genes in all of the three datasets were identified to be significant for prognosis prediction (Fig. 1A). An overview of the 52 genes with the corresponding hazard ratio for OS in TCGA was displayed (Fig. 1B). The 52 genes were employed in the LASSO Cox regression model to develop a prognostic signature for OS using TCGA as a discovery cohort. The partial likelihood deviance test selected the optimal 14 genes, and the corresponding coefficients were generated at the optimal log λ of -3.68 (Fig. 1C and 1D). Hence, the risk score for the individual patient was calculated as follow: risk score = CDO1_exp_ × 0.01+G6PD_exp_ × 0.08+PPARG_exp_ × 0.02+ FABP4_exp_ × 0.04+LDHA_exp_ × 0.26+CCL20_exp_ × 0.04+SPP1_exp_ × 0.03+RORC_exp_ × (-0.06)+HDAC1_exp_ × 0.24+STC2_exp_ × 0.09+HDGF_exp_ × 0.20+EPO_exp_ × 0.09+GPD1L_exp_ × 0.05+IL18RAP_exp_ × (-1.20). Patients were divided into two groups with low risk and high risk according to the median of the risk score. PCA was further conducted to determine the clustering ability of risk score. It showed that patients in two subgroups were distinctively clustered by the 14 -gene signature in all three datasets (Fig. 1E). The clinical characteristics of patients grouped by different risks were listed in Supplementary Table 1. The relationship of risk score and pathological staging was explored, and it showed that patients in the advanced stage had significantly higher risk scores in all of the three cohorts (Fig. S1).

### Prognostic value of the redox-immune signature

**Figure 2:**
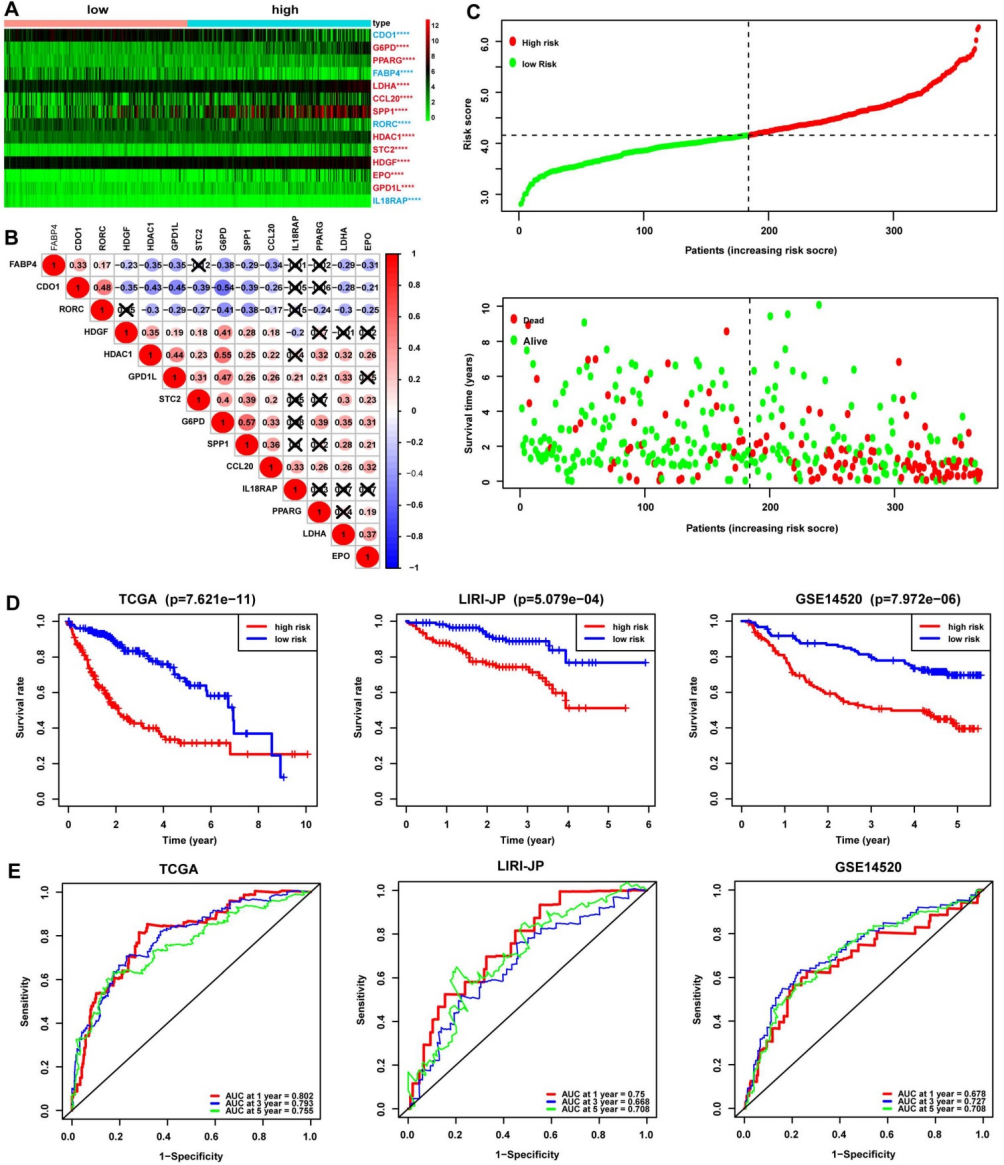
** Assessment of the prognostic value of the 14-gene signature in TCGA, LIRI-JP, and GSE14520 cohorts.** (A) Expression profile of the 14 signature genes grouped by different risk in TCGA. The font in blue represented the relatively low expression of the gene in the high-risk group, while the font in red represented a relatively high expression of the gene compared to the low-risk group. (B) Spearman correlation analysis of the 14 signature genes in TCGA. The number in the circle represented the correlation coefficient. The blue circle referred to a negative correlation, and the circle in red referred to a positive correlation, and the symbol “⮾” referred to no correlation. (C) Risk curve depicting the distribution of risk score and survival time. (D) Kaplan-Meier plot of OS by risk group in TCGA, LIRI-JP, and GSE14520 cohort, respectively. (E) ROC curve of the 14-gene signature for 1, 3, and 5-year OS prediction in TCGA, LIRI-JP, and GSE14520 cohort, respectively.

The redox-immune signature consisted of 4 RRGs, including CDO1, G6PD, LDHA, GPD1L, and 10 IRGs named PPARG, FABP4, CCL20, SPP1, RORC, HDAC1, STC2, HDGF, EPO, IL18RAP. The expression pattern of the 14 signature genes was shown in the heatmap (Fig. 2A). The DEGs analysis demonstrated that CDO1, FABP4, RORC, and IL18RAP were downregulated and G6PD, PPARG, LDHA, CCL20, SPP1, HDAC1, STC2, HDGF, EPO, and GPD1L were upregulated in the high-risk group compared with those in the low-risk group (Fig. 2A). Additionally, correlation analysis showed either a positive or negative correlation in most of the signature genes (Fig. 2B). The risk curve suggested seemingly more patients at dead status with increasing risk scores in TCGA cohorts (Fig. 2C). Furthermore, Kaplan-Meier analysis was performed in TCGA, LIRI-JP, and GSE14520 cohorts to evaluate the signature's prognostic value. The results revealed significantly worse survival in patients with high risk than those with low risk (Fig. 2D). ROC curves were then plotted to estimate the prediction accuracy of the signature. AUCs at 1-, 3-, 5-year in TCGA were 0.802, 0.793, and 0.755, respectively, hinting at an excellent predictive value of the signature, further validated in LIRI-JP and GSE14520 cohorts (Fig. 2E).

### Strong power for prognosis prediction of the redox-immune signature

**Figure 3:**
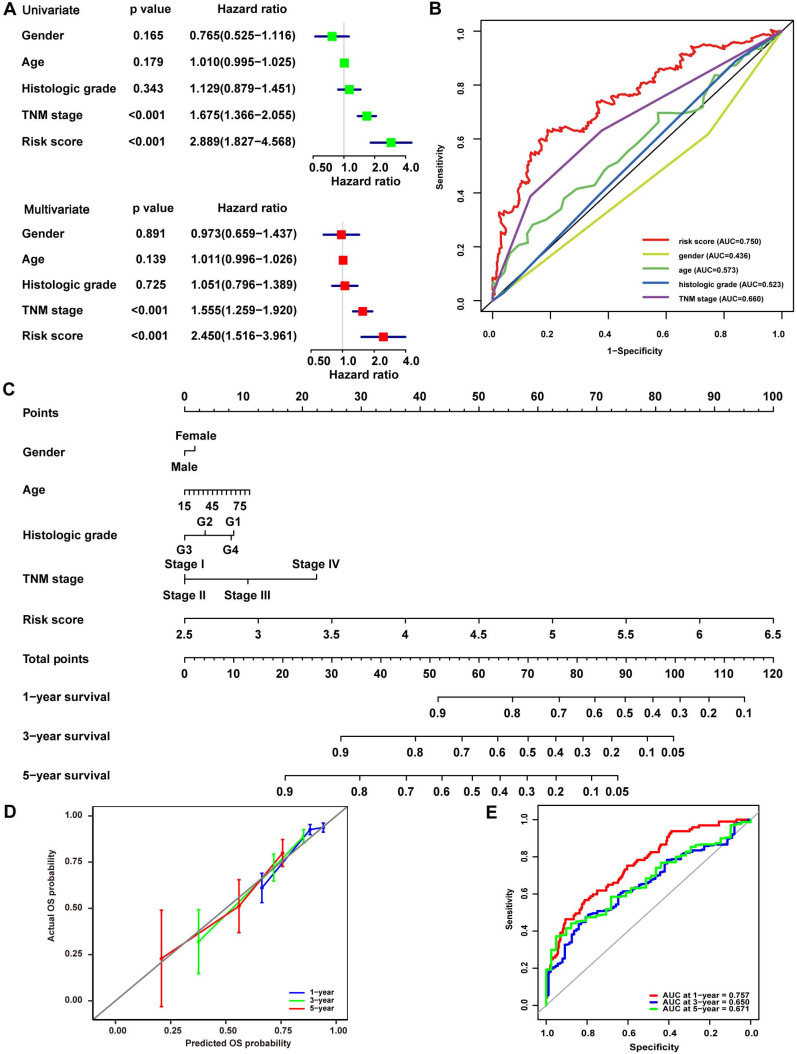
** Validation of the prognostic value of the 14-gene signature in TCGA.** (A) Univariate and multivariate Cox analyses evaluating the prognostic value of risk score regarding OS in TCGA. (B) ROC curve of risk score and other clinical parameters for 5-year OS prediction in TCGA. (C) Nomogram constructed by the 14-gene signature for 1, 3, and 5-year OS prediction in TCGA. (D) Calibration curve of the nomogram for 1, 3, and 5-year OS prediction in TCGA. The x-axis referred to predicted survival while the y-axis referred to the observed survival, and the gray line represented perfect prediction. (E) ROC curve of the nomogram for 1, 3, and 5-year OS prediction in TCGA.

Univariate and multivariate Cox analyses were conducted in the TCGA cohort to illustrate the signature's prognostic value further. In the Univariate Cox analyses, more advanced TNM stage (HR, 1.675; 95% CI, 1.366-2.055; P<0.001) and higher risk score (HR, 2.889; 95% CI, 1.827-4.568; P<0.001) were significantly associated with poor OS in HCC (Fig. 3A). In the multivariate Cox analyses, more advanced TNM stage (HR, 1.555; 95% CI, 1.259-1.920; P<0.001) and higher risk score (HR, 2.450; 95% CI, 1.516-3.961; P<0.001) were identified as independent risk factors for OS (Fig. 3A). ROC curves were drawn to compare these parameters' prediction accuracy, and it declared that the AUC of the risk score was 0.750, while the AUC of the TNM stage was 0.660, indicating a better prognostic value of risk score than TNM stage (Fig. 3B). These findings were then confirmed in LIRI-JP cohort, while the AUC of risk score was slightly lower than that of TNM stage and BCLC stage in GSE14520 cohort (Fig. S2), such variance among the three cohorts may be related to cohort heterogeneity. Nomogram integrating risk score and other clinical variables including gender, age, histological grade, and TNM stage for 1-, 3-, and 5-year OS prediction was established using the TCGA cohort (Fig. 3C). The calibration curves estimating OS probability showed a significant agreement of the predicted OS probability with the observed OS probability, implicating the nomogram's excellent performance for predicting OS (Fig. 3D). The C-index of the nomogram was 0.756, and the AUCs at 1-, 3-, and 5-year of the nomogram were 0.757, 0.650, and 0.671, respectively (Fig. 3E), suggesting good discrimination ability when combining C-index with AUC. The performance of the nomogram was further validated in LIRI-JP cohort and GSE14520 cohort, with a C-index of 0.761 and the AUCs at 1-, 3-, and 5-year of 0.788, 0.742, 0.675 in LIRI-JP cohort (Fig. S3A-B), and a C-index of 0.736 and AUCs at 1-, 3-, and 5-year of 0.777, 0.779, 0.671 in GSE14520 cohort (Fig. S3C-D).

### Metabolic regulation associated with redox markers

**Figure 4:**
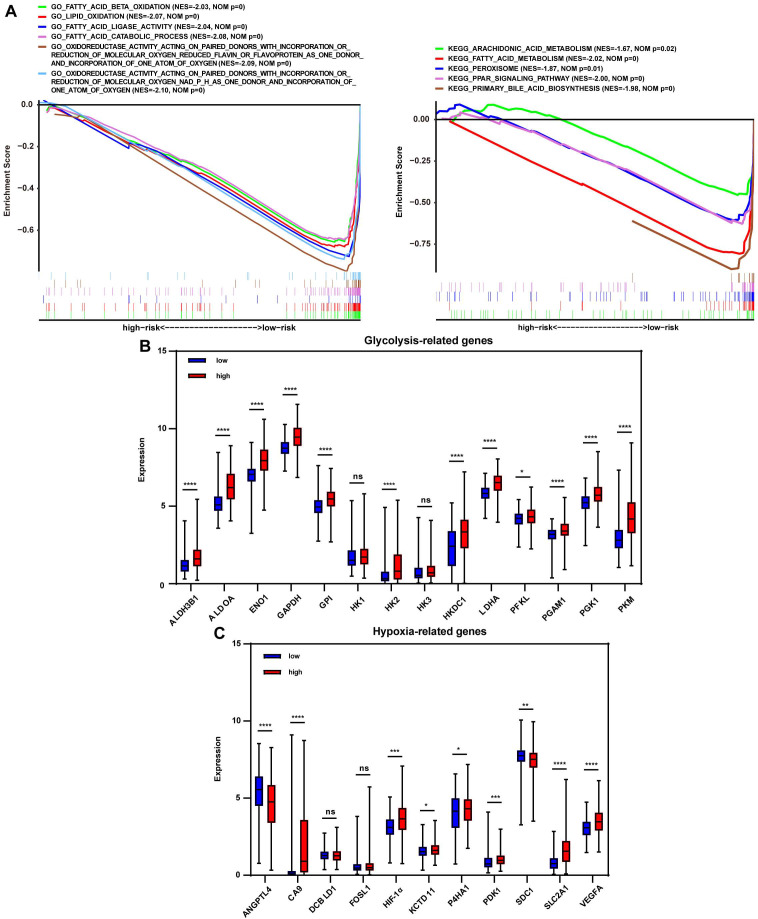
** Redox-related alterations are analyzed by GSEA and gene differential analysis in TCGA.** (A) GO and KEGG enrichment analyses of redox-related pathways ranked in the top 30 in the risk-based group. (B) Expression of glycolysis-related genes in the low-risk and high-risk group. (C) Expression of hypoxia-related genes in the low-risk and high-risk group. * p < 0.05, ** p < 0.01, *** p < 0.001, **** p < 0.0001, ns p > 0.05.

To investigate the signature-based prognostic classifier's mechanism, GO and KEGG terms associated with risk stratification were explored by GSEA. GO terms related to oxidoreductase activity were enriched in the low-risk group in TCGA, LIRI-JP, and GSE14520 cohort, respectively (Fig. 4A, Fig. S4A, and Fig. S5A), inferring that more active regulation of redox homeostasis was involved in the low-risk group. The redox state plays a crucial role in metabolic reprogramming 12. Metabolic alterations were explored in this study. GSEA found that lipid consumption related pathways, such as fatty acid beta-oxidation, fatty acid catabolic process, and fatty acid ligase activity, were strongly involved in the low-risk group in the TCGA cohort, LIRI-JP and GSE14520 cohorts (Fig. 4A, Fig. S4A, and Fig. S5A). Additionally, the key glycolysis-related markers (ALDH3B1, ALDOA, ENO1, GAPDH, GPI, HK1, HK2, HK3, HKDC1, LDHA, PFKL, PGAM1, PGK1 and PKM) were compared between the subgroups. It showed that expressions of the majority of genes, except HK1 and HK3, were significantly higher in the high-risk group (Fig. 4B), indicating increased glycolysis activity in the high-risk group, which were further documented in LIRI-JP and GSE14520 cohorts (Fig. S4B and Fig. S5B). This finding was consistent because glycolysis-related enzymes such as LDHA were highly expressed in the high-risk group (Fig. 2A). For KEGG analysis, peroxisome was enriched in the low-risk group and pathways closely associated with peroxisome function, such as peroxisome proliferator-activated receptor (PPAR) signaling pathway, fatty acid metabolism, and primary bile acid biosynthesis (Fig. 4A). A trend for similar enrichments was observed in the LIRI-JP cohort but not in the GSE14520 cohort (Fig. S4A and Fig. S5A). Expressions of hypoxia-related genes reported in a previous study were evaluated to reflect the hypoxia condition in the subgroups since that peroxisome plays a vital role in modulating oxygen concentration 21, 22. HIF-1α, CA9, KCTD11, PDK1, SLC2A1, and VEGFA levels were significantly higher in the high-risk group (Fig. 4C), suggesting a severe hypoxic microenvironment in the high-risk group. These findings were further verified in LIRI-JP and GSE14520 cohorts (Fig. S4C and Fig. S5C).

### Immune regulation associated with immune markers

**Figure 5:**
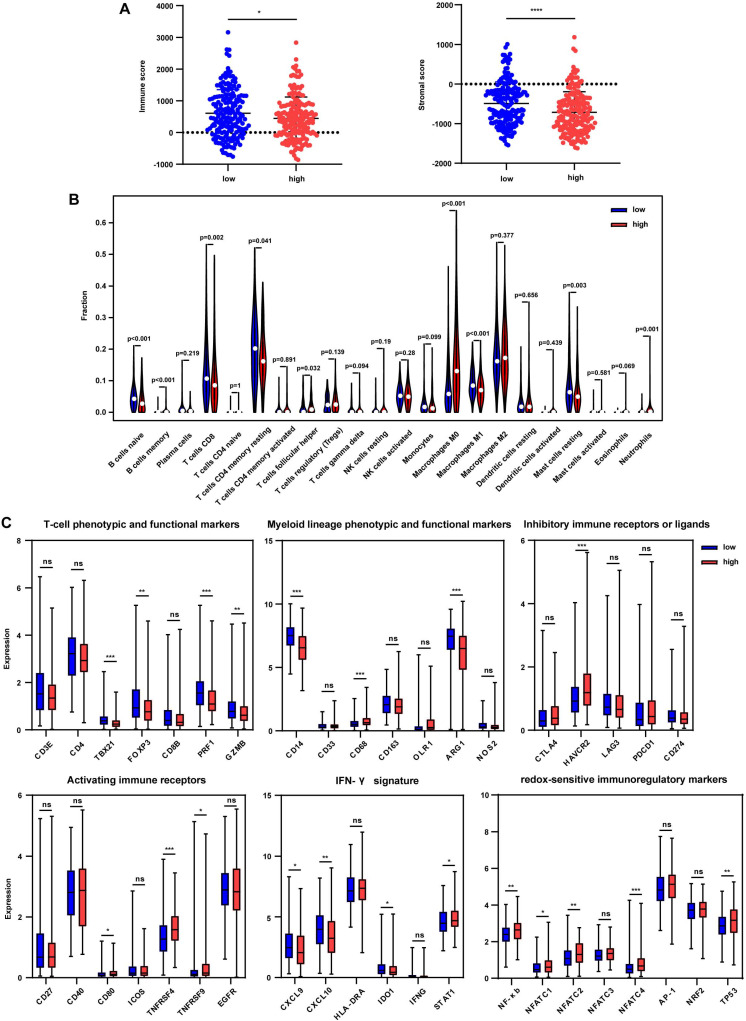
**Tumor immune microenvironment in the TCGA cohort grouped by different risks.** (A) Immune score and stromal score in the low-risk and high-risk group. (B) Comparison of tumor-infiltrating immune cells between two groups. (C) Expression of immune-related genes in the low-risk and high-risk group. * p < 0.05, ** p < 0.01, *** p < 0.001, **** p < 0.0001, ns p > 0.05.

The tumor immune microenvironment was further estimated to uncover the effect of immune signature-based prognostic classification. ESTIMATE method was used to calculate the immune score and stromal score, and results showed that the immune score was significantly decreased in the high-risk group in the TCGA cohort (Fig. 5A). However, no differences were found between the subgroups in LIRI-JP and GSE14520 cohorts (Fig. S6A and S6B). Besides, significantly lower stromal scores were observed in the high-risk group in TCGA and GSE14520 cohorts but not in the LIRI-JP cohort (Fig. 5A, Fig. S6A, and S6B). These together documented that both immune cells and non-immune cells were less infiltrated in tumor bed in the high-risk group. To better elaborate on the immune microenvironment, the CIBERSORT algorithm was employed to evaluate the abundance of 22 immune cells using the TCGA cohort. Patients in the high-risk group presented significantly lower infiltration of anti-tumor immune cells, including naïve B cells, memory B cells, CD8+ T cells, and M1 macrophages, while a higher fraction of pro-tumor immune cell, neutrophils (Fig. 5B). Furthermore, the GSE14520 cohort analysis showed that anti-tumor immune cells, including γδT cells and M2 macrophages, were decreased in the high-risk group, despite no appealing findings in exploring the LIRI-JP cohort (Fig. S6C and S6D). It seemed controversial that M2 macrophages were downregulated in the high-risk group in GSE14520 cohort since that the pro-tumor role of M2 macrophages in HCC supposed an upregulation of itself in the high-risk group 23. However, immune microenvironment-related tumor process is regulated by the synergistic effect of varieties of immune cells far more than M2 macrophages 24. Then, immune-related markers belonging to the specific subset were analyzed in the TCGA cohort. The results showed that T-cell phenotypic and functional markers, namely TBX21, FOXP3, PRF1, and GZMB, were significantly downregulated in the high-risk group (Fig. 5C). Regarding myeloid lineage phenotypic and functional markers, CD14 and ARG1 expressions were decreased in the high-risk group. Simultaneously, CD68 was highly expressed in the high-risk group, indicating a lower percentage of monocytes and M2 macrophages and a higher abundance of M1 macrophages (Fig. 5C). Paradoxically, the results were discrepant with that from CIBERSORT analysis since that single marker could not accurately reflect the percentage of specific cells. Inhibitory immune marker (HAVCR2) and activating immune receptors (CD80, TNFRSF4, and TNFRSF9) were both at higher levels in the high-risk group, suggesting a complex immune microenvironment within the tumor (Fig. 5C). IFN-γ markers, including CXCL9, CXCL10, IDO1, were decreased in the high-risk group while STAT1 was increased (Fig. 5C). To further uncover the relationship between redox and immunity, expressions of redox-sensitive immunoregulatory factors were compared. It showed that NF-κb, NFATC1, NFATC2, NFATC4, and TP53 levels were higher in the high-risk group, implicating that probably more severe oxidative stress in the high-risk group upregulated these redox sensors to orchestrate an adaptive response.

## Discussion

Although exciting progress has been made in immune therapy for HCC recently, drug resistance occurs in many patients due to the inter- and intra-tumor heterogeneity 25. Immune therapy combining with drugs targeting angiogenesis, such as sorafenib and bevacizumab, improve the response and prognosis of HCC 26, 27. Therefore, a signature established by the union of different cancer hallmarks would devote to the patient's classification, which may benefit prognosis prediction and development of targeted treatment tactics. Oxidative stress is a trait of many cancers, including HCC, which plays a crucial role in regulating metabolism, cell proliferation, and immune response in HCC 28. Here, we developed a 14-gene signature based on redox and immune-related markers for prognosis prediction of HCC. The signature showed good performance for predicting 1-, 3-, 5-year survival in the three cohorts. However, it was worth noting that there was a little difference in the sensitivity among the 1-, 3-, 5-year AUC curve, perhaps the increasing lost to follow-up rate or uncontrolled confounding factors over time leading to analysis bias that were responsible for such variance. We also addressed the redox-related metabolic alterations and immune cell infiltration to elucidate the distinct prognosis mechanism in the subgroups.

Oxidoreductase activity was highly enriched in the low-risk group, indicating a more robust buffering to the imbalance between the production of oxidative species and antioxidant molecules. It is speculated that a better prognosis of patients in the low-risk group partly resulted from the more effective manipulation of redox disorder, considering that redox perturbation generally acts as a driver in carcinogenesis 10, 29. Metabolic orchestrating is intimately coupled to redox signaling to meet the enormous energy consumption and high proliferation rate of cancer cells 6. Metabolic pathways, such as lipid oxidation, peroxisomes, and bile acid biosynthesis, were observed to correlate with low risk. Consistently, a study characterizing the heterogeneous redox responses in HCC revealed that subjects with high survival tend to overexpress genes involved in metabolic and energy regulation (e.g., electron transport chain, fatty acid metabolism, amino acid metabolism, bile acid and bile salt transport)^30^. Peroxisomes are dynamic cellular organelles where fatty acid β-oxidation and bile acid metabolism mainly occur 31, 32. Peroxisomes control the production and detoxifying of free radicals to maintain the redox hemostasis 33. PPAR signaling pathway, which can be activated by the products of fatty acid oxidation, was also enriched in the low-risk group. It seems controversial about the pro-tumor or anti-tumor role of peroxisomes in cancer for either increased or decreased abundance of peroxisomes observed in distinct cancers 34-36. In HCC, a series of researches have validated the inverse correlation between peroxisomes and tumor growth and tumor grade 37-39. These support our findings that peroxisomes related pathways were enriched in the low-risk group, indicating the tumor suppressor role of peroxisomes in HCC.

The intricate interplay between redox signaling and energy metabolism is confirmed in human cancers 6, 12. Our results suggested abundant glycolysis activity in the high-risk group. Cancer cells tend to rely more on glycolysis instead of OXPHOS to acquire energy quickly and benefit to cancer progression, even if in the presence of oxygen, which is known as the “Warburg effect” 40. Elevated ROS shifts OXPHOS to glycolysis by directly or indirectly regulating various metabolic enzymes' activity 6. Metabolic shaping induced alterations in the production of oxidoreductase molecules like NADPH and ROS disturb redox homeostasis in turn, making up a metabolic-redox circuit 6. In this study, the violent redox dysregulation in the high-risk group was probably responsible for the increased glycolysis and the poor prognosis. Hypoxia condition was evaluated because peroxisomes dynamically affect oxygen concentration by consuming and producing O_2_ 21, 22. Our results exhibited severe hypoxia in the high-risk group, indicated by the relatively high expression of hypoxia-related genes including HIF-1α. Actually, a research by R. Benfeitas et al. stratifying HCC patients into G6PD cluster and ALDH2 cluster basing on the differential expression pattern of redox genes demonstrated that G6PD-clustered genes were associated with hypoxic behavior and poor survival compared with ALDH2 cluster, and HIF-1α was part of the G6PD cluster acting as an unfavorable factor 30, this together with our results suggested close link between redox and hypoxia, and the crucial role of such link in tumor progression. Studies have confirmed that excessive ROS is in favor of HIF-1α stabilization, in turn, which promotes the transcription of hypoxia-related genes such as VEGF 41, 42. Comprehensively, perhaps hypoxia microenvironment and excessive ROS in the high-risk group jointly enhanced the expression of hypoxia-related genes that accelerated tumor progression.

Immune infiltration was investigated to validate our model further. Immune cell abundance was decreased in the high-risk group. Moreover, lower infiltrations of anti-tumor immune cells, including naïve B cells, memory B cells, CD8+ T cells, and M1 macrophages, while higher fractions of pro-tumor immune cells, like neutrophils, were presented in the high-risk group. Tumor microenvironments such as hypoxia, low PH, and lactate also instigate an immunosuppressive network that helps cancer cells fulfill immune evasion 43. In this study, severe hypoxia and glycolysis-induced lactate accumulation, low PH may be linked to the immunosuppressive microenvironment in the high-risk group. On the other hand, aberrant ROS drives the activation of multiple redox-sensitive immunoregulatory transcription factors, such as NF-κb, NFAT, and AP-1 44, 45, and induces cytokines secretion, consequently modulating T cell activation and shifting T cell phenotypes 13. Our results confirmed higher expressions of NF-κb, NFATC1, NFATC2, NFATC4, and TP53 in the high-risk group, indicating more violent oxidative stress and corresponding rewiring of immune infiltration.

Overall, active response to redox perturbation in the low-risk group facilitated immune defense against cancer cells. In contrast, the failure of effective regulation on redox signaling in the high-risk group led to a relatively immunosuppressive microenvironment. For patients in the high-risk group, strategies targeting redox signaling or redox-related alterations such as glycolysis and hypoxia combining with subsequent immune therapy may be beneficial. To make it more convictive, the basic experiments by constructing of animal model to explore the interplay between redox and immune, as well as the effect of such factors on tumor growth was considerable in the future.

## Supplementary Material

Supplementary figures and tables.
