# Supplementary material for: Identification and validation of redox-immune based prognostic signature for hepatocellular carcinoma
Source: Int J Med Sci. 2021 Mar 10;18(9):2030–41. doi: 10.7150/ijms.56289 (PMC8040390; doi:10.7150/ijms.56289)
Supplement: Supplementary file 1 — Supplementary figures and tables. [file ijmsv18p2030s1.pdf]

Supplementary Table 1 Baseline characteristics of patients in the discovery and validation cohorts

**TCGA**	Whole cohort (n=368)	Low risk (n=184)	High risk (n=184)	P value
Gender				0.0946
Male	249	132	117	
Female	119	52	67	
Age				0.2489
<60	165	77	88	
≥60	203	107	96	
Histologic grade				<0.0001
G1+G2	231	135	96	
G3+G4	132	46	86	
NA	5	3	2	
TNM stage				0.0002
I+II	257	145	112	
III+IV	87	27	60	
NA	24	12	12	
Status				<0.0001
Alive	237	144	93	
Dead	131	40	91	
**LIRI-JP**	Whole cohort (n=231)	Low risk (n=116)	High risk (n=115)	P value
Gender				0.8504
Male	170	86	84	
Female	61	30	31	
Age				0.1435
<60	45	27	18	
≥60	186	89	97	
TNM stage				0.0131
I+II	141	80	61	
III+IV	90	36	54	
Status				0.0012
Alive	188	104	84	
Dead	43	12	31	
**GSE14520**	Whole cohort (n=242)	Low risk (n=121)	High risk (n=121)	P value
Gender				0.0344
Male	211	100	111	
Female	31	21	10	
Age				0.1124
<60	192	91	101	
≥60	50	30	20	
BCLC stage				0.0030
0+A	172	98	74	

B+C	53	17	36	
NA	17	6	11	
TNM stage				0.0027
I+II	174	99	75	
III+IV	51	16	35	
NA	17	6	11	
Status				0.0002
Alive	146	87	59	
Dead	96	34	62	

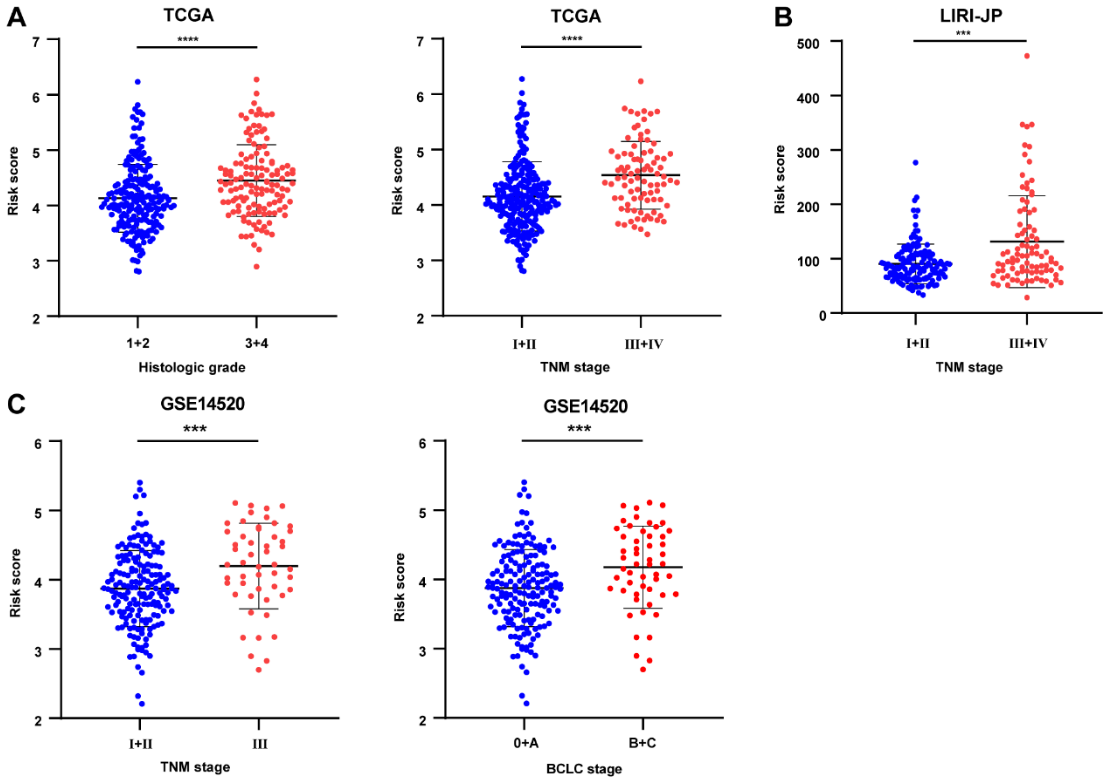

Figure S1 Relationship between risk score and clinical parameters in TCGA, LIRI-JP, and GSE14520 cohorts. (A) Comparison of the risk score in TCGA grouped by histologic grade and TNM stage, respectively. (B) Comparison of the risk score in LIRI-JP grouped by the TNM stage. (C) Comparison of the risk score in GSE14520 grouped by TNM stage and BCLC stage, respectively. *** $p < 0.001$, **** $p < 0.0001$.

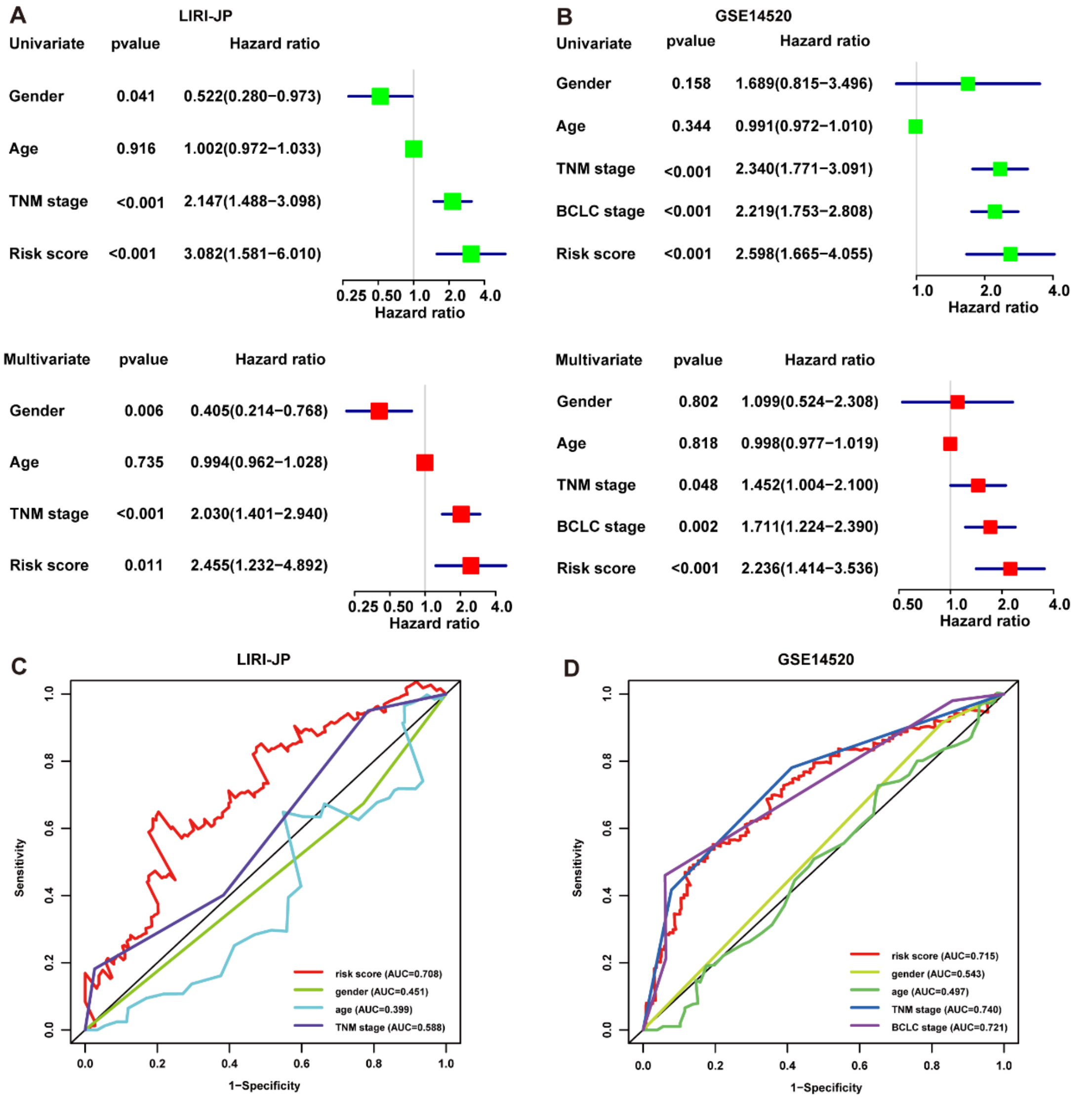

Figure S2 Prognostic value of risk score in LIRI-JP and GSE14520 cohorts. (A and B) Univariate and multivariate Cox analyses evaluating the prognostic value of risk score regarding OS in LIRI-JP and GSE14520, respectively. (C and D) ROC curve of the risk score for 5-year OS prediction in LIRI-JP and GSE14520, respectively.

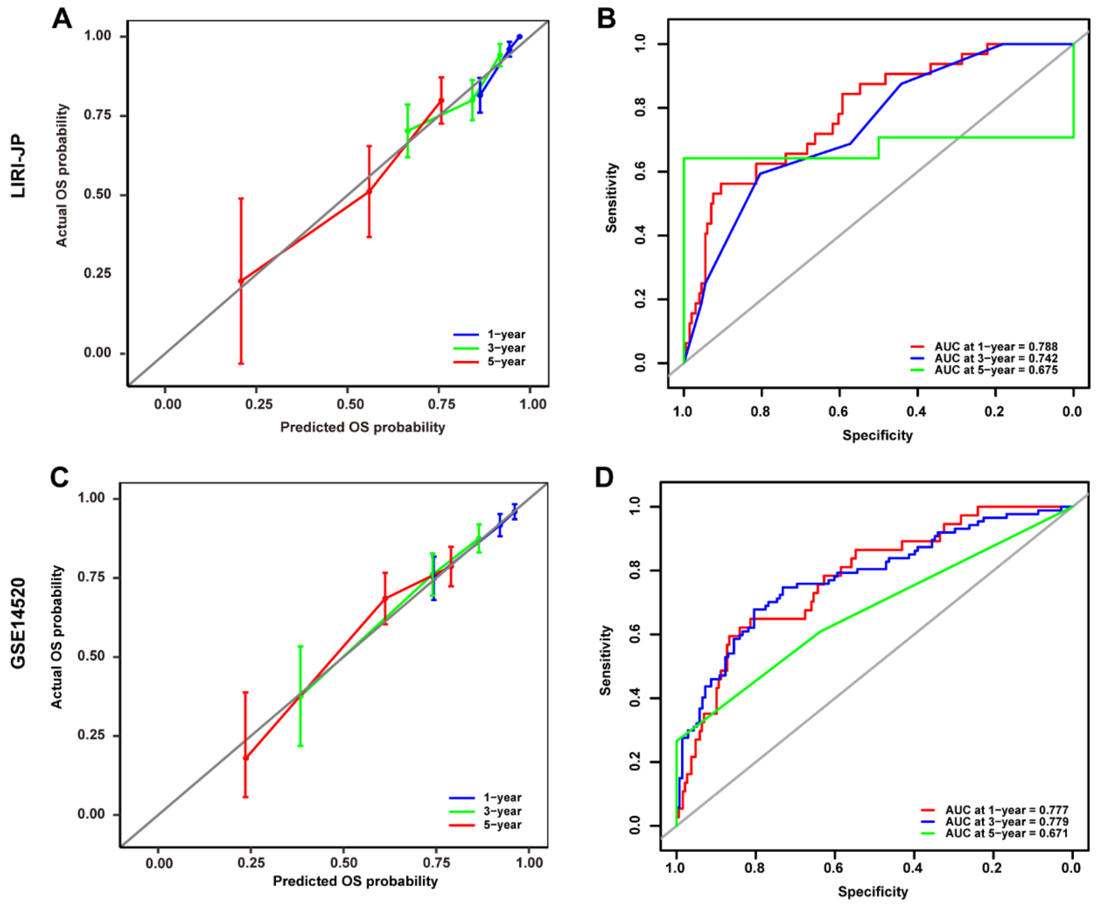

Figure S3 Prognostic value of nomogram in LIRI-JP and GSE14520 cohorts. (A and C) Calibration curve of the nomogram for 1, 3, and 5-year OS prediction in LIRI-JP and GSE14520 cohort, respectively. The x-axis referred to predicted survival while the y-axis referred to the observed survival, and the gray line represented perfect prediction. (B and D) ROC curve of the nomogram for 1, 3, and 5-year OS prediction in LIRI-JP and GSE14520 cohort, respectively.

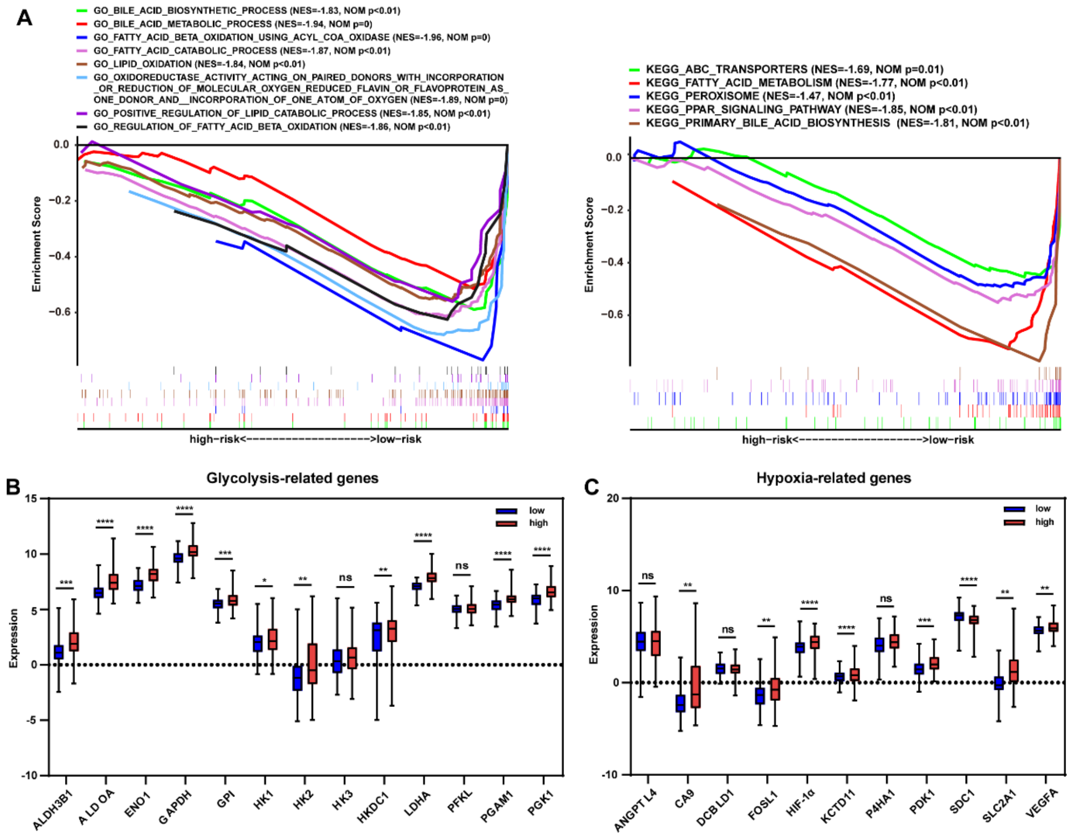

Figure S4 Redox-related alterations are analyzed by GSEA and gene differential analysis in LIRI-JP. (A) GO and KEGG enrichment analyses of redox-related pathways ranked in the top 30 in the risk-based group. (B) Expression of glycolysis-related genes in the low-risk and high-risk group. (C) Expression of hypoxia-related genes in the low-risk and high-risk group. * $p < 0.05$, ** $p < 0.01$, *** $p < 0.001$, **** $p < 0.0001$, ns $p > 0.05$.

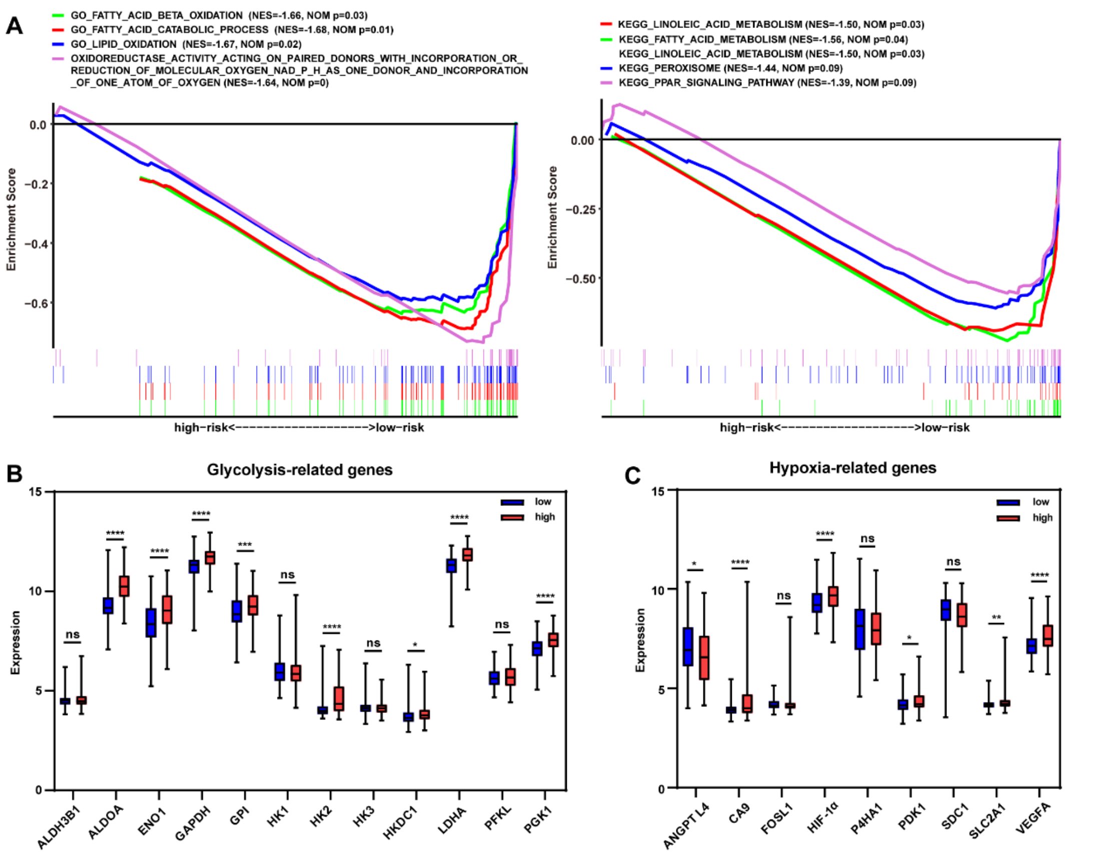

Figure S5 Redox-related alterations are analyzed by GSEA and gene differential analysis in GSE14520. (A) GO and KEGG enrichment analyses of redox-related pathways ranked in the top 30 in the risk-based group. (B) Expression of glycolysis-related genes in the low-risk and high-risk group. (C) Expression of hypoxia-related genes in the low-risk and high-risk group. * $p < 0.05$, ** $p < 0.01$, *** $p < 0.001$, **** $p < 0.0001$, ns $p > 0.05$.

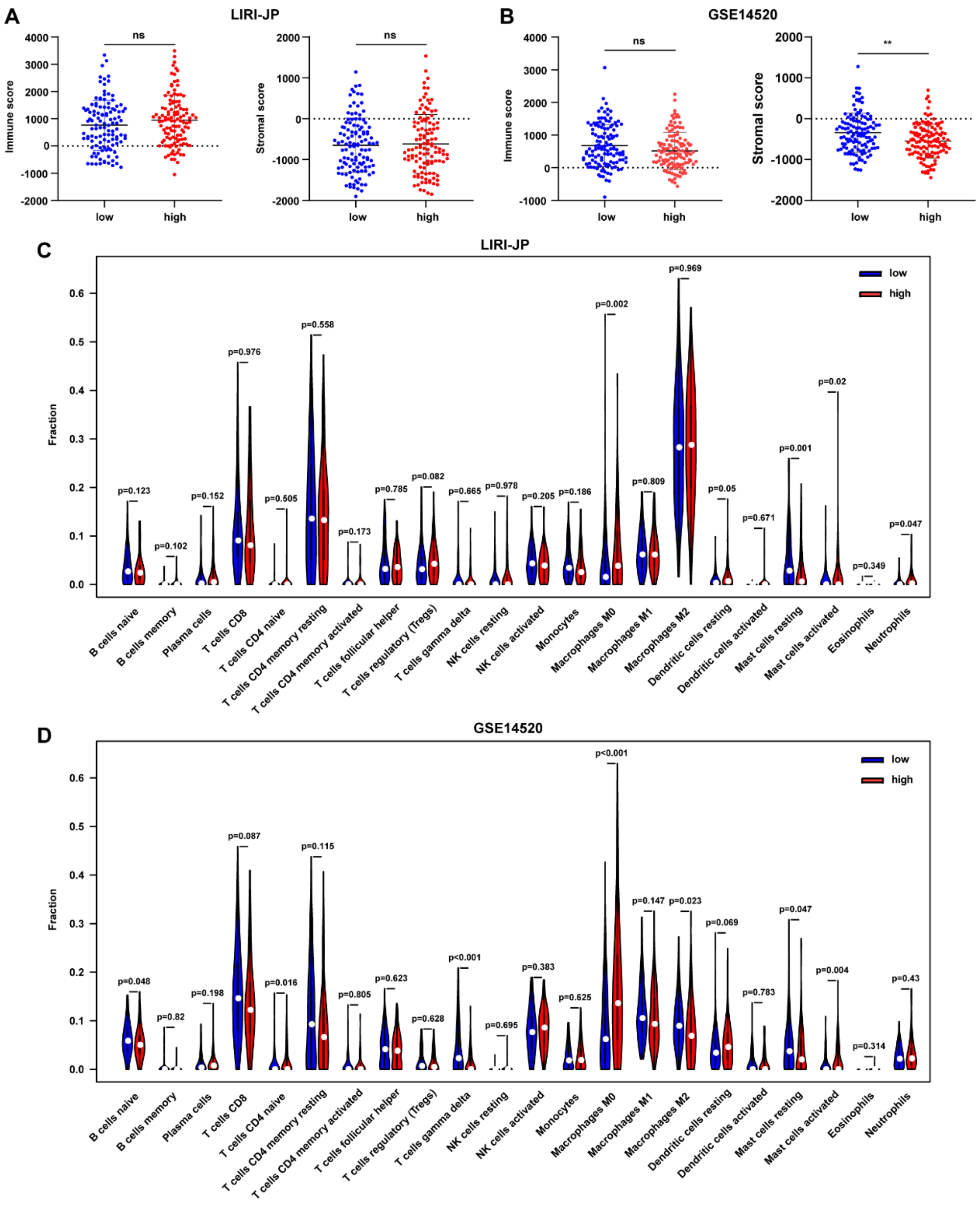

Figure S6 Tumor immune microenvironment in LIRI-JP and GSE14520 cohorts grouped by different risks. (A and B) Immune score and stromal score in LIRI-JP and GSE14520, respectively. (C and D) Comparison of tumor-infiltrating immune cells between two groups in LIRI-JP and GSE14520, respectively. ** p < 0.01, ns p > 0.05.
